# Deficient Production of Reactive Oxygen Species Leads to Severe Chronic DSS-Induced Colitis in Ncf1/p47^phox^-Mutant Mice

**DOI:** 10.1371/journal.pone.0097532

**Published:** 2014-05-29

**Authors:** Tiago Rodrigues-Sousa, Ana Filipa Ladeirinha, Ana Raquel Santiago, Helena Carvalheiro, Bruno Raposo, Ana Alarcão, António Cabrita, Rikard Holmdahl, Lina Carvalho, M. Margarida Souto-Carneiro

**Affiliations:** 1 ImmunoMetabolic Pharmacology Group, CNC- Centro de Neurociências e Biologia Celular, Universidade de Coimbra, Coimbra, Portugal; 2 Departamento de Anatomia Patológica, Faculdade de Medicina, Universidade de Coimbra, Coimbra, Portugal; 3 Instituto Biomédico de Investigação da Luz e Imagem, Faculdade de Medicina, Universidade de Coimbra, Coimbra, Portugal; 4 Medical Inflammation Research, Karolinska Institute, Stockholm, Sweden; 5 Departamento de Patologia Experimental, Faculdade de Medicina, Universidade de Coimbra, Coimbra, Portugal; Goethe Universität Frankfurt, Germany

## Abstract

**Background:**

Colitis is a common clinical complication in chronic granulomatous disease (CGD), a primary immunodeficiency caused by impaired oxidative burst. Existing experimental data from NADPH-oxidase knockout mice propose contradictory roles for the involvement of reactive oxygen species in colitis chronicity and severity. Since genetically controlled mice with a point-mutation in the *Ncf1* gene are susceptible to chronic inflammation and autoimmunity, we tested whether they presented increased predisposition to develop chronic colitis.

**Methods:**

Colitis was induced in Ncf1-mutant and wild-_type_ mice by a 1^st^ 7-days cycle of dextran sulfate sodium (DSS), intercalated by a 7-days resting period followed by a 2^nd^ 7-days DSS-cycle. Cytokines were quantified locally in the colon inflammatory infiltrates and in the serum. Leukocyte infiltration and morphological alterations of the colon mucosa were assessed by immunohistochemistry.

**Results:**

Clinical scores demonstrated a more severe colitis in Ncf1-mutant mice than controls, with no recovery during the resting period and a severe chronic colitis after the 2^nd^ cycle, confirmed by histopathology and presence of infiltrating neutrophils, macrophages, plasmocytes and lymphocytes in the colon. Severe colitis was mediated by increased local expression of cytokines (IL-6, IL-10, TNF-α, IFN-γ and IL-17A) and phosphorylation of Leucine-rich repeat kinase 2 (LRRK2). Serological cytokine titers of those inflammatory cytokines were more elevated in Ncf1-mutant than control mice, and were accompanied by systemic changes in functional subsets of monocytes, CD4^+^T and B cells.

**Conclusion:**

This suggests that an ineffective oxidative burst leads to severe chronic colitis through local accumulation of peroxynitrites, pro-inflammatory cytokines and lymphocytes and systemic immune deregulation similar to CGD.

## Introduction

Mutations in the components of the NADPH oxidase 2 (NOX2) complex compromise the normal oxidative burst by phagocytes and B cells and are related to hyper-inflammation phenomena and recurrent opportunistic infections in patients with chronic granulomatous disease (CGD) and several animal models of inflammatory diseases [1]–[5]. Since the majority of CGD patients present mutations in the gp91^phox^/Cybb subunit (about 70%) or in the p47^phox^/Ncf1 subunit (about 20%) of NOX2, several studies have focused on understanding the immune response in corresponding animal models [6]–[9]. In particular, a point mutation in the *Ncf1* gene of B10.Q mice impairs the production of reactive oxygen species (ROS) leading to increased susceptibility to infections with *Staphylococcus xylosus*, *Staphylococcus aureus* and *Burkholderia cepacia* [10], and to the development of severe chronic autoimmune disorders such as collagen-induced arthritis and experimental autoimmune encephalopathy [11]–[13]. These hyper-inflammatory responses are directly related to the incapacity of producing ROS, since the administration of oxidants or reestablishing the burst capacity of macrophages with a functional *Ncf1* lead to permanent recovery in treated animals [8], [11]–[14]. In knockout mice for either *Cybb* or *Ncf1*, an increased susceptibility to infections and to chronic inflammation was observed [6]–[9]. Since bowel inflammation, resembling Crohn's disease (CD), is a common complication of CGD, with patients presenting elevated titers of Crohn's associated antibodies even in the absence of active colitis [15], *gp91^phox−/−^*, *Ncf1^−/−^* and –more recently- *p40^phox−/−^* mice have been used to understand how the lack of ROS-production associates with colitis [19]–[21]. However, the results in *Ncf1^−/−^* and *gp91^phox−/−^* mice were not consistent with those observed in CGD patients, since dextran sulfate sodium (DSS)-induced colitis was indistinguishable between *Ncf1^−/−^* mice and their wild type (WT) counterparts, whereas in *gp91^phox−/−^* mice DSS-induced colitis was milder than in the WT groups [16]. Furthermore, these studies focused on a single-round of colitis induction, without addressing the possibility of differences in repeatedly challenged mice. Contrasting to the studies on *Ncf1^−/−^* and *gp91^phox−/−^* mice, when DSS-colitis was induced in *p40^phox−/−^* mice the colon tissue was more injured in the mice deficient of ROS production than in the control group. This was associated with a more severe disease and poor recovery [17]. However, since Ncf4 interacts directly with Ncf2, knocking out *p40^phox^* might induce a broader phenomenon, making it difficult to assess which is the unique contribution of *p40^phox^* to colitis severity. By inducing two cycles of colitis with DSS intercalated by a resting period in BQ.*Ncf1^m1J^* mice with a point mutation in the *Ncf1* gene, similar to that in CGD patients, we propose a new colitis model to study how the lack of oxidative burst can lead to the development of chronic inflammatory bowel disease (IBD).

## Materials and Methods

### Animals

6–8 weeks old male homozygous Ncf1-mutant BQ.*Ncf1^m1J/m1J^* (Ncf1*, n = 15) and wild-type (WT, n = 15) BQ mice (C57BL/10 expressing H-2^q^) were obtained from breeding heterozygous mice, as previously described [14]. Animals were bred and maintained under standard conditions, with food and water *ad libitum* in a specific pathogen–free environment. All procedures were done under anesthesia in order to reduce discomfort and stress during sample collection. All animal studies were approved by the internal CNC Ethics Committee, and were in accordance with EU legislation for experimental animal welfare.

### Induction of colitis

**Figure 1:**
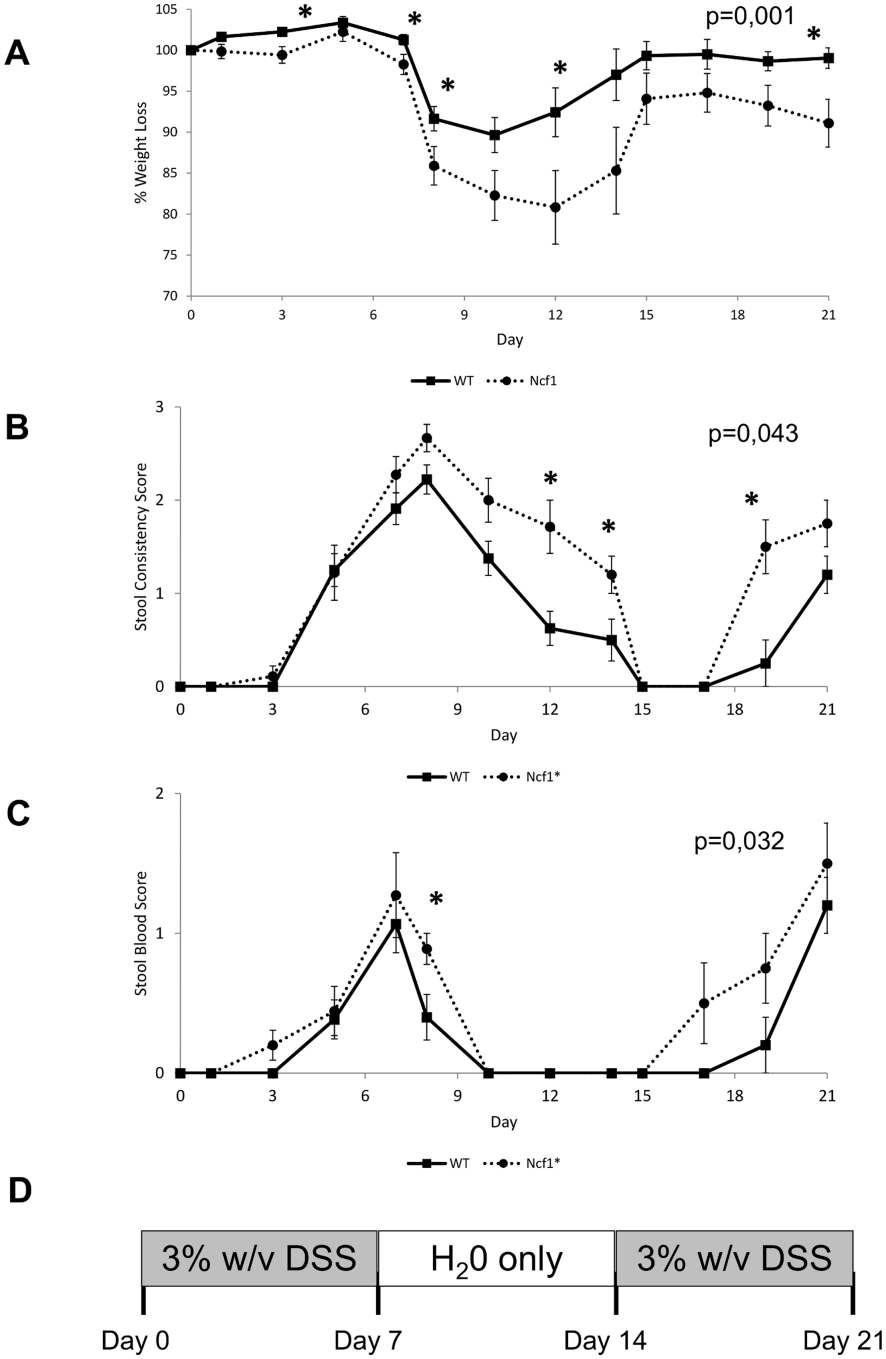
Clinical scores of colitis are more severe in Ncf1* mice. A) Changes in average weight ± SE, (baseline weight: WT = 25.8±0.5 g, Ncf1* = 26.5±0.8 g). B) Changes in stools consistency ± SE. C) Presence of blood in stools ± SE. Asterisks indicate p<0.05, Mann-Whitney test between Ncf1* and WT. P-values on the top right corner indicate the statistical difference between curves, permutation test. D) Schematic overview of the DSS treatment plan showing the induction and recovery periods.

Colitis was induced by oral administration of 3% (w/v) DSS (average 40,000 g/mol, AppliChem, Darmstadt, Germany) *ad libitum* in drinking water for 2 cycles. The induction protocol consisted of 7 days of treatment with DSS, followed by 7 days of resting on normal water, and a second 7 days cycle with DSS (Fig. 1D). Five Ncf1* and WT mice were sacrificed at the end of each time point: day 7; day 14; and day 21, for histopathologic assessment of colitis and blood collection.

### Clinical Evaluation

The clinical scores of colitis: weight change, diarrhea, colorectal bleeding and survival were monitored every other day. Blood scoring: 0- no blood; 1- visible blood; 2- rectal bleeding. Consistency scoring: 0- Normal; 1- Soft but formed; 3- very soft; 4- diarrhea. For all clinical scores the number of mice evaluated at each cycle was for both groups: Days 0 to 7 n = 15, Days 8 to 14 n = 10; Days 15 to 21 n = 5.

### Histopathological evaluation of colitis

Separate swiss-rolls of distal, transverse and proximal colon were fixed in 4% neutral buffered formalin and stained with hematoxylin/eosin (HE) according to standard protocols. Additionally, immunohistochemistry was performed on proximal colon sections. In brief, endogenous peroxidase activity was quenched by 15 minutes incubation with 3% diluted hydrogen peroxide. Nonspecific binding was blocked with Ultra V Block (Ultra Vision Kit; TP-125-UB; Lab Vision Corporation; Fremont CA; USA). Incubation with primary antibodies against B220 (Instituto Gulbenkian de Ciência, Oeiras, Portugal), CD11b (clone Mac1, Instituto Gulbenkian de Ciência, Oeiras, Portugal) and CD3 (polyclonal, DakoCytomation, Glostrup, Denmark) to detect B cells, macrophages and T cells, respectively, or against FoxP3, IL-6, IL-10, IL-17A, IFN-γ, TNF-α (all from Biolegend) and Dundee-MJFF LRRK2 PhosphoSer935 (phosphorylated Leucine-rich repeat kinase 2; clone UDD2 10(12), RabMab/Abcam) was followed by incubation with biotin-labeled secondary antibody (Ultra Vision Kit; TP-125-BN; Lab Vision Corporation; Fremont CA; USA). Primary antibody binding was localized in tissues using peroxidase-conjugated streptavidin (Ultra Vision Kit; TP-125-HR; Lab Vision Corporation; Fremont CA; USA) and 3,3-diaminobenzidine tetrahydrochloride (RE7190-K; Novocastra Laboratories Ltd, Newcastle, United Kingdom) was used as chromogen, according to manufacturer's instructions. Hematoxylin was used to counterstain the slides. Morphological alterations and infiltration by inflammatory cells were screened by two pathologists in a blinded fashion.

Inflammation was scored for each colon section according to the number of inflammatory foci present: 0- no inflammatory focus; 1- one inflammatory focus; 2- two inflammatory foci; 3- three or more inflammatory foci. Dysplasia was scored for each colon section using a semi-quantitative scale: 0- no dysplasia; 1- nuclear pluristratification and hypercromatic independent glands; 2- nuclear pluristratification and hypercromatic independent glands and complex glands; 3- nuclear pluristratification and hypercromatic complex glands with scattered mitosis. The infiltration of lymphocytes, neutrophils and plasmocytes was scored based on cell morphology, and corresponded to the percentage of each subset within the total inflammatory cells. Infiltration of B220^+^, CD3^+^ or Mac-1^+^ cells was scored based on the immunohistochemistry cell-specific staining as 0 (absence of cell type); + (<50% positive cells); ++ (50–75% positive cells) and +++ (>75% positive cells), considering the total inflammatory infiltrate in each case using the web-based ImmunoMembrane image-analysis software (http://153.1.200.58:8080/immunomembrane/).

Relative quantification of cytokines and pLRRK2 was performed using the web-based ImmunoRatio image-analysis software (http://153.1.200.58:8080/immunoratio/).

### Superoxide anion detection

Formalin-fixed, paraffin-embedded sections (4 µm thick) were mounted on polysine-coated glass slides. Sections were deparaffinized in xylene and rehydrated in PBS. The sections were incubated for 90 min at 37°C with the fluorescent probe dihydroethidium (DHE, 5 µmol/l; Molecular Probes, Life Technologies, USA). In the presence of superoxide anions, DHE is oxidized to ethidium, which intercalates with DNA, and yields bright red fluorescence. After washing with PBS, sections were incubated with 4′,6-diamidino-2-phenylindole (DAPI) for 20 min. Sections were rinsed in PBS and mounted on glass slides using Glycergel mounting medium (Dako, Denmark). From each section, four random sections were acquired in a laser scanning confocal microscope (Zeiss LSM 710, Germany). The specificity of the staining was evaluated by the omission of the dye (negative control). Densitometric analysis of DHE fluorescence intensity was performed using ImageJ Software 1.46r (NIH, USA).

### Immunofluorescence detection of nitrotyrosine

The presence of 3-nitrotyrosine residues, an indirect marker of peroxynitrite formation, was evaluated by immunofluorescence. After deparaffinization, antigen retrieval was performed using citrate buffer at 95°C. sections were incubated with 1% bovine serum albumin (BSA) in PBS (PBA) for 1 h, followed by incubation with 2% normal goat serum in PBA for 30 min. Sections were then incubated overnight at 4°C with anti-nitrotyrosine antibody (1∶100; Merck Millipore, USA) prepared in PBA. After washing, sections were incubated for 1 h with Alexa Fluor 488 goat anti-rabbit secondary antibody (1∶200, Life Technologies) prepared in PBA. After washing, nuclei were stained with DAPI. Slides were mounted with Glycergel. A negative control without primary antibody was performed. From each section, four random sections were acquired in a laser scanning confocal microscope (Zeiss LSM 710, Germany). Densitometric analysis of 3-nitrotyrosine residues fluorescence intensity was performed using ImageJ Software 1.46r (NIH, USA).

### Serum cytokine quantification

Serum samples were collected at each time-point. Cytokine titers in the sera were quantified using the cytometric bead arrays, mouse Th1/Th2/Th17/Th22 13plex FlowCytomix Multiplex kit (Bender MedSystems, Austria) according to the manufacturer's instructions and analyzed with FlowCytomix Pro Software (eBioscience, San Diego, USA). Concentrations of cytokines below the limit of detection of the assay were given zero value.

### Leukocyte Phenotyping

Peripheral blood samples were collected from the base of the tail on days 0, 7, 14 and 21. Mononuclear cells were stained in a standard method using fluorochrome-conjugated anti-mouse monoclonal antibodies against: CD3, CD4, CD8, CD25, CD69, CXCR5, IgM, CD19, IgD, CD107a, CD27, CD62L, NK1.1, CD11b, and Ly-6c (all from Biolegend). Intracellular staining was done after saponin permeabilization using fluorochrome-conjugated anti-mouse FoxP3 and CD68 (all from Biolegend). Irrelevant, directly conjugated, murine IgG1 or IgG2 (Biolegend) were used to ascertain background staining. 25000 events were collected within the lymphocyte gate. After calibration with CST beads, single-fluorochrome stained cells were used for instrument compensation and PMT setup. All samples were analyzed on a BD FACSCanto II (Becton Dickinson) and data were analyzed with FlowJo 7.6.4 software (Tree Star).

### Statistical Analysis

All data were tested for normal distribution with Levene's test. Since data did not follow a normal distribution the non-parametric Kruskal-Wallis test followed by a non-parametric Mann-Whitney test were used to compare values between groups and time-points, using Statview 5.0.1 software (SAS Institute Inc, USA). Statistical differences between curves were determined using a 2-sided hypothesis permutation test with 10000 permutations [18] (http://bioinf.wehi.edu.au/software/russell/perm/). Differences were considered significant for p<0, 05.

## Results

### Clinical signs of DSS-induced colitis

Throughout the whole observation period the Ncf1* mice lost significantly more weight than the WT (p = 0.001, between curves, Fig. 1A). On day 8, after the first treatment cycle with DSS, both animal groups presented significant (p<0.05) weight loss ranging from 10–15% as compared to baseline (baseline weight: WT = 25.8±0.5 g, Ncf1* = 26.5±0.8 g). For the WT group the weight-loss continued until day 10, after which they recovered, reaching their baseline weight by day 15, which was kept until the end of the study. In contrast, Ncf1* mice continued to lose weight until day 12, recovered up till 95% of their initial weight between days 14 and 17, and started to lose again thereafter, thus having only in average 90% of their initial body-weight at the end of the second cycle of DSS administration.

The presence of blood in stools and the consistency of the feces are two further clinical signs of DSS-induced colitis. Throughout the various phases of the study, Ncf1* mice presented significantly worst clinical scores for the presence of blood (p = 0.032, between curves) and consistency (p = 0.043, between curves) of stools than WT (Fig. 1B and 1C). Throughout the resting period the Ncf1* group scored worst for the presence of blood in stools and stool consistency, and their recovery was delayed when compared to the WT animals.

### Histopathological assessment of DSS-Induced Colitis

**Figure 2:**
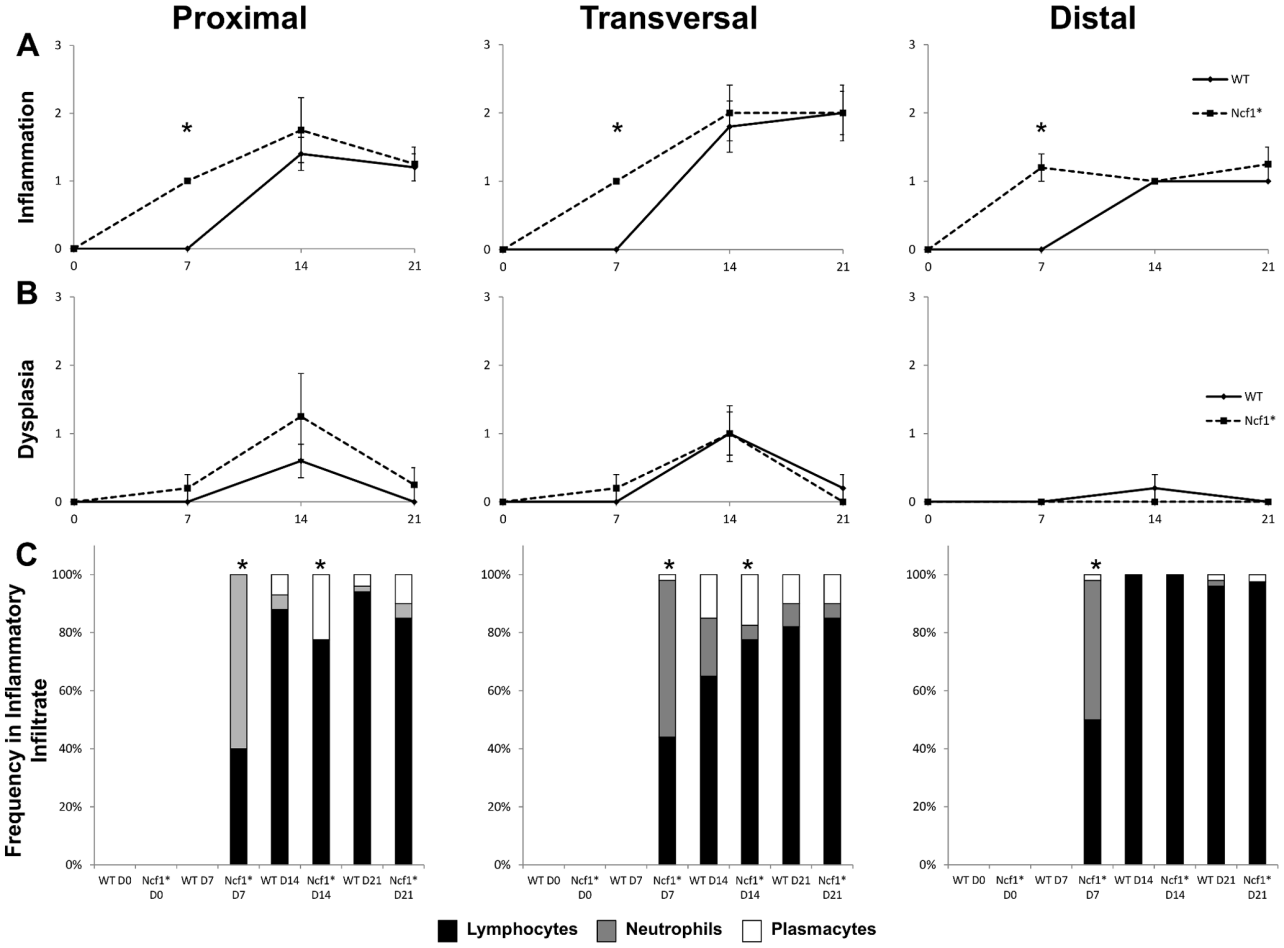
Histological evaluation shows that Ncf1* mice develop a severe chronic colitis with several inflammatory foci and dysplasia, particularly in the proximal and transversal sections of the colon, with a skewed leukocyte distribution. A) Inflammatory score in the different sections of the colon throughout the experimental period. B) Dysplasia score in the different sections of the colon throughout the experimental period. C) Distribution of the leukocytes subsets in the different sections of the colon throughout the experimental period. Asterisks indicate p<0.05, Mann-Whitney test between Ncf1* and WT. Inflammation and dysplasia scoring system is detailed in the methods section.

**Figure 3:**
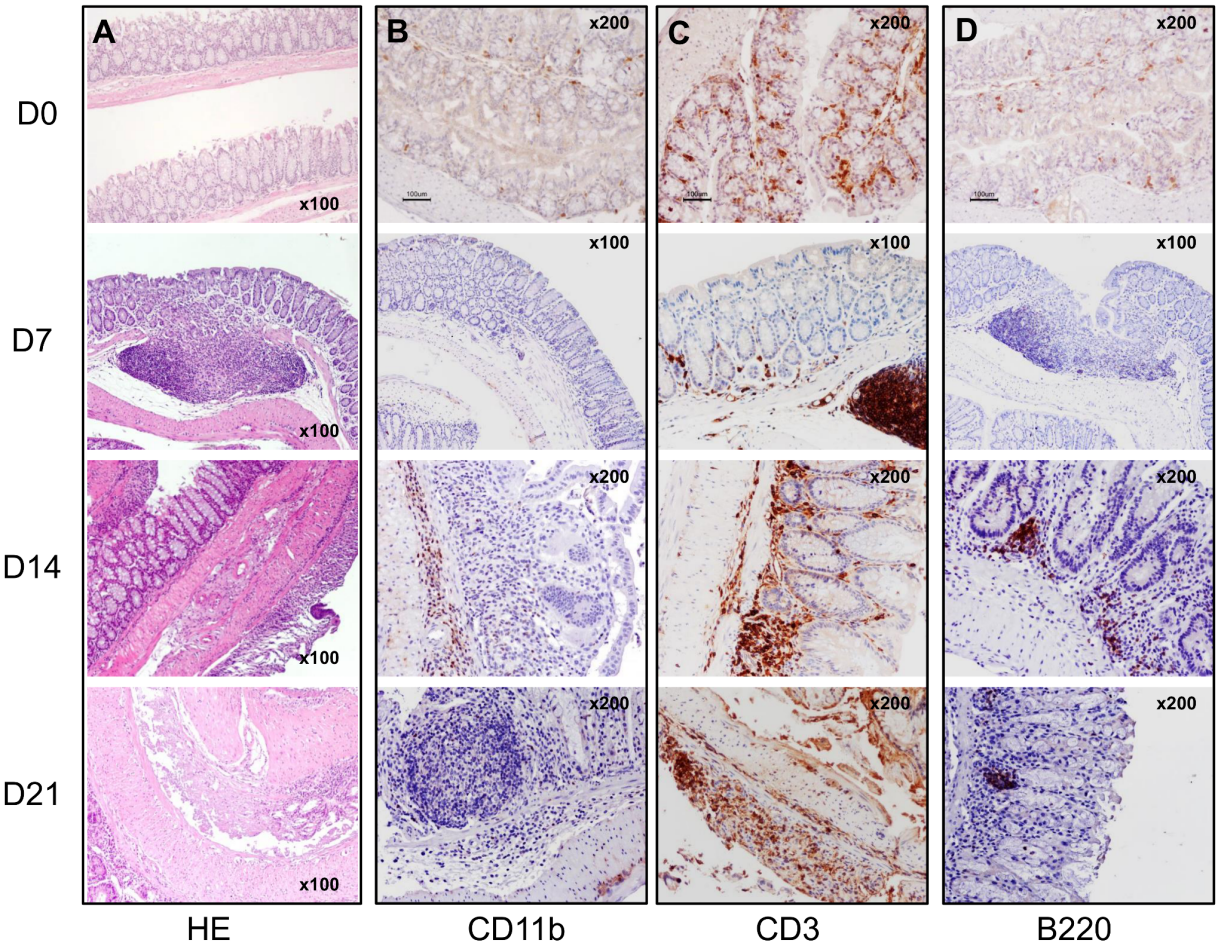
Histological and immunohistochemistry analyses of the WT colon show erosions, epithelial hyperplasia and low grade dysplasia, with progressive infiltration of CD3^+^ T and B220^+^ B lymphocytes and Mac-1^+^ cells. A) Hematoxylin-eosin (HE) staining of the colon during the different phases of the DSS-induced colitis protocol. B) Immunohistochemistry of Mac-1^+^ cells in the inflammatory infiltrates at days 0, 7, 14 and 21. C) Immunohistochemistry of CD3^+^ cells in the inflammatory infiltrates at days 0, 7, 14 and 21. D) Immunohistochemistry of B220^+^ cells in the inflammatory infiltrates at days 0, 7, 14 and 21. Magnification inserted in the images.

**Figure 4:**
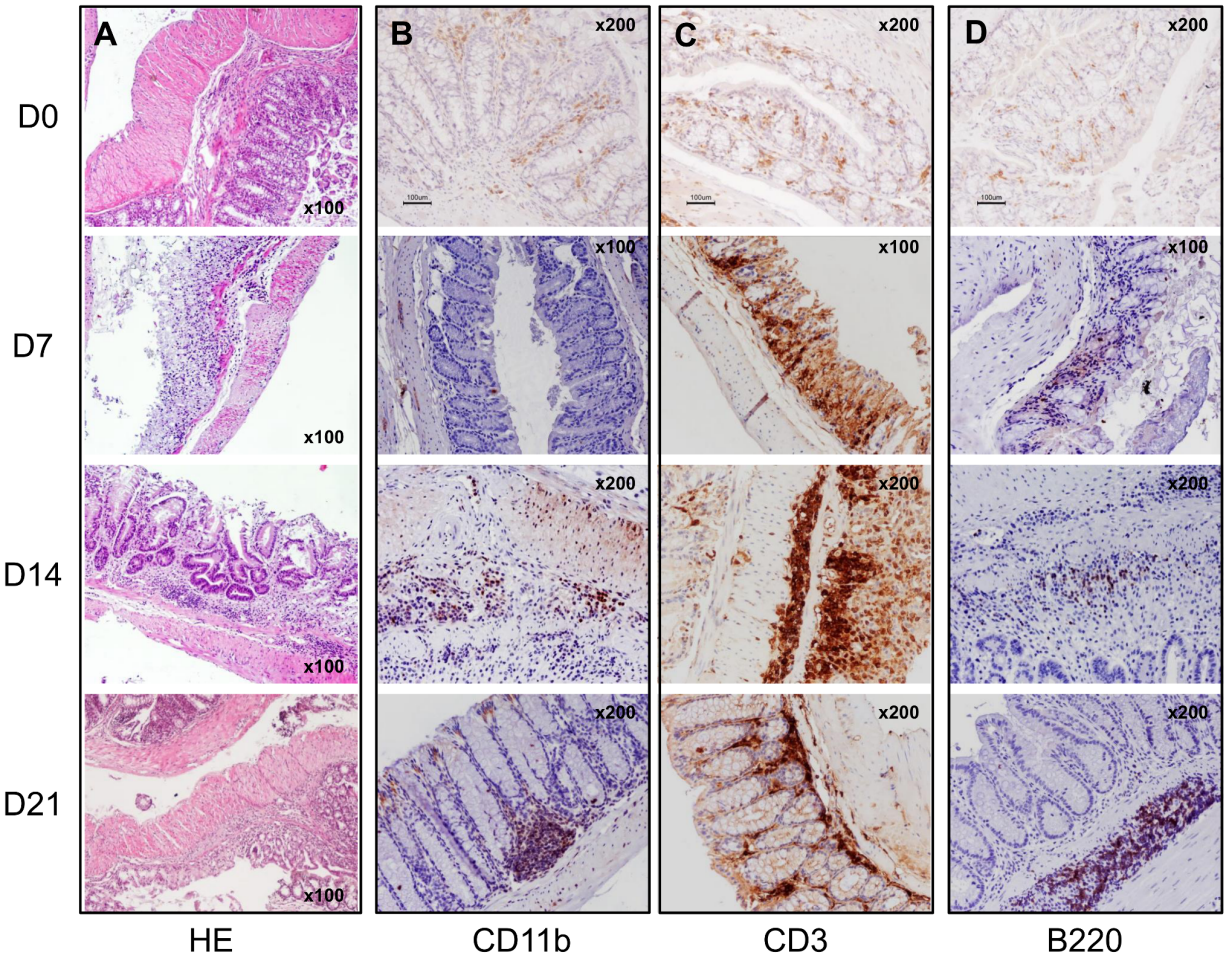
Histological and immunohistochemistry analyses of the Ncf1* colon show ulcers, epithelial hyperplasia and severe dysplasia, with massive infiltration of CD3^+^ lymphocytes, and subsequently of B220^+^ B lymphocytes and Mac-1^+^ cells in the later time-points. A) Hematoxylin-eosin (HE) staining of the colon during the different phases of the DSS-induced colitis protocol. B) Immunohistochemistry of Mac-1^+^ cells in the inflammatory infiltrates at days 0, 7, 14 and 21. C) Immunohistochemistry of CD3^+^ cells in the inflammatory infiltrates at days 0, 7, 14 and 21. D) Immunohistochemistry of B220^+^ cells in the inflammatory infiltrates at days 0, 7, 14 and 21. Magnification inserted in the images.

**Figure 5:**
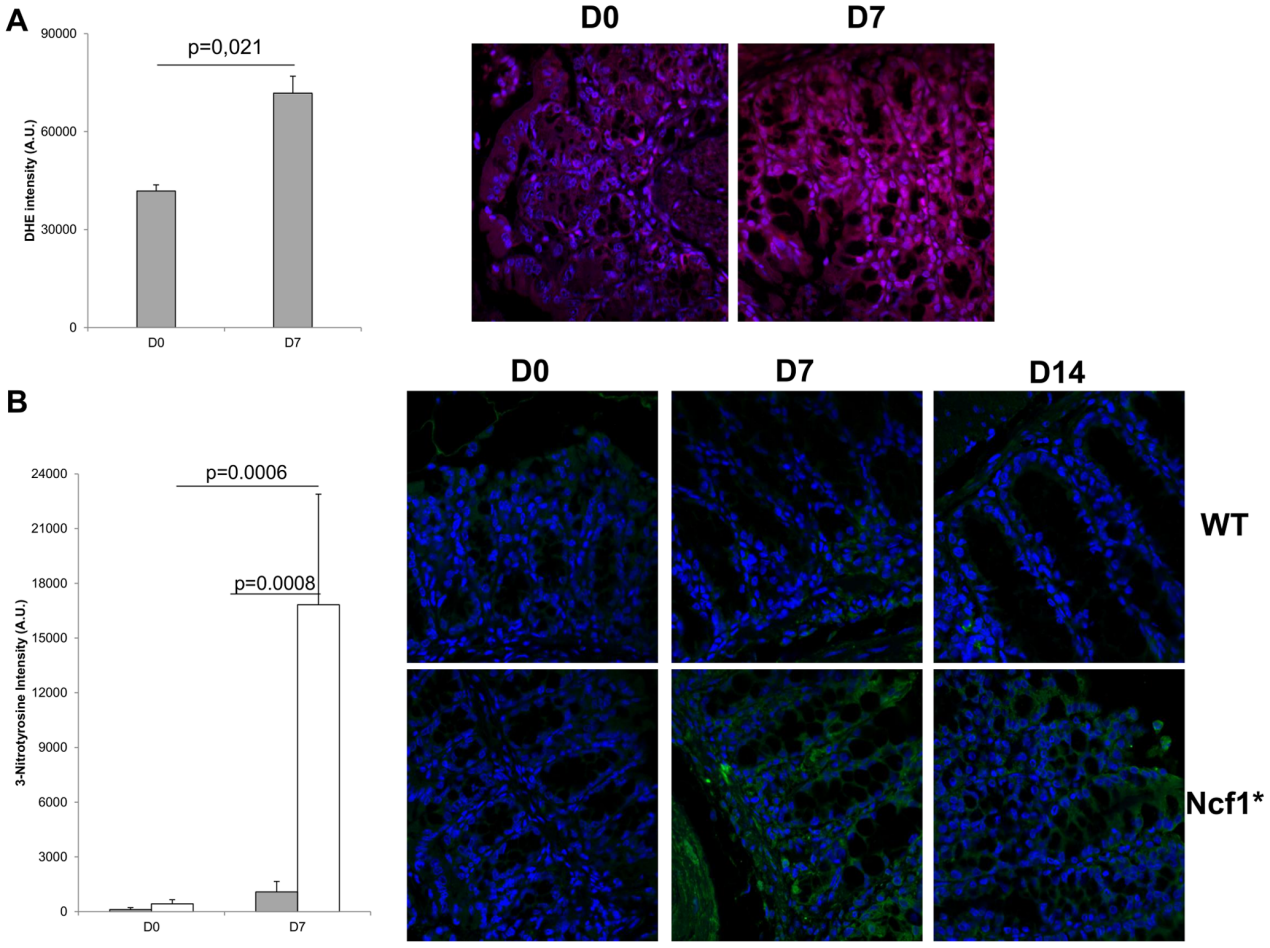
ROS levels increase in WT mice colonic inflammatory infiltrates after colitis induction, whereas Ncf1* mice increase the local production of peroxynitrites, which remains high after the recovery period. A) Relative DHE fluorescence intensity in colon sections of WT mice, Mann-Whitney test. B) Representative fluorescence immunohistochemistry of local ROS (red) production in WT mice before and after colitis induction (magnification 400×). C) Relative 3-nitrotyrosin residues fluorescence intensity in colon sections of WT (grey bars) and Ncf1* mice (white bars), Mann-Whitney test. D) Representative fluorescence immunohistochemistry of peroxynitrites (green) production in the colonic inflammatory infiltrates in WT and Ncf1* mice (magnification 400×).

The baseline (D0) HE staining from colon sections shows that the *Ncf1*-mutation alone does not induce alterations in the colon architecture nor detectable inflammatory spots when compared to WT animals (D0 in Fig. 2A, 3A, 4A). In both groups there were scattered resident CD3^+^ (score: 0) and B220^+^ lymphocytes (score: 0), and CD11b^+^ macrophages (score: 0) (D0 in Fig. 3B–D and 4B–D). By the time the first cycle of DSS-administration was over (D7) evident differences could be seen in the colonic morphology of both groups when compared to D0. In WT mice neither inflammation (Fig. 2A) nor dysplasia (Fig. 2B) could be observed, even though there was low grade erosion of the colonic mucosa (Fig. 3A), and a significant increase in local ROS production (Fig. 5A, B). This contrasted to the findings in Ncf1* mice, which in addition to erosion and immature fibroblasts presented significantly more inflammation (Fig. 2A) in all colon sections. This inflammatory infiltrate was mainly composed of neutrophils and CD3^+^ T lymphocytes (score: +++), though some plasmocytes were equally present (Fig. 2C, Fig. 4D). Additionally, at D7 the Ncf1* mice had a significantly higher local production of peroxynitrites in the colonic inflammatory infiltrates than at D0 or comparing to the WT group (Fig. 5C, D). After the resting period at D14, the colon tissue of the WT mice presented erosion, epithelial hyperplasia with mitosis and nuclear atypia (Fig. 3A), low grade dysplasia (Fig. 2B) and an inflammatory infiltrate mainly composed of CD3^+^ (score: +++) and B220^+^ lymphocytes (score: ++), a few CD11b^+^ macrophages (score: +), granulocytes (score: +) and plasmocytes (score: +) (Figs. 2A & C, 3B–E). The Ncf1* mice presented a more severe injury of the colonic tissue, with ulcers reaching the *muscularis propria* (Fig. 4A), severe dysplasia with small back to back glandules with atypical mitoses and anisocarioses (Figs. 2B, 4A), and the composition of the inflammatory infiltrate was mainly CD3^+^ (score: +++) and B220^+^ lymphocytes (score: ++), CD11b^+^ macrophages (score: ++), plasmocytes (score: +), and a reduced amount of granulocytes (less than 5%) (Figs. 2C, 4B–E). The local production of peroxynitrites in Ncf1* colon inflammatory infiltrates remained higher than in the WT animals (Fig. 5D). After the second cycle of colitis induction, D21, the colon of WT mice had ulcers, and epithelial regeneration with glandular hyperplasia without nuclear atypia (Fig. 3), and the inflammatory infiltrate was mostly comprised of CD3^+^ lymphocytes (score: +++), a few CD11b^+^ macrophages (score: +), and less than 10% granulocytes and plasmocytes (score: 0 to +) (Figs. 2C, 3B, C & D). The Ncf1* mice equally presented ulcers with re-epithelialization (Fig. 4A), however the epithelial regeneration showed nuclear atypia and low focal dysplasia (Fig. 2B). Moreover, the inflammatory infiltrate was mainly composed of CD3^+^ lymphocytes (score: +++) and CD11b^+^ macrophages (score: ++), plasmocytes (score: +) and less than 5% granulocytes (Figs. 2C, 4B, C & E). The presence of FoxP3^+^ T cells was equivalent for both groups in all time points (data not shown).

### Systemic changes in functional leukocyte subsets

**Figure 6:**
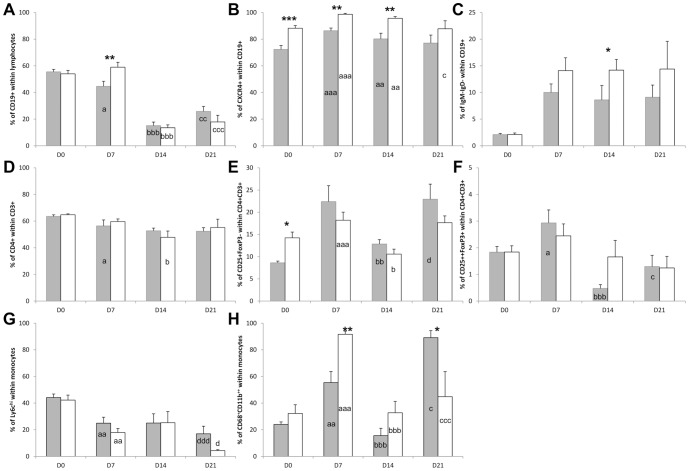
Analysis of peripheral blood functional leukocyte subsets at the different phases of DSS-colitis induction shows altered frequencies of circulating B and CD4^+^ T lymphocytes and CD11b^+^ monocytes between Ncf1* and WT mice. A) Frequency of CD19^+^ B lymphocytes within total lymphocytes. B) Frequency of CXCR4^+^ cells within CD19^+^ B lymphocytes. C) Frequency of IgM^−^IgD^−^ cells within CD19^+^ B lymphocytes. D) Frequency of CD4^+^ T lymphocytes within CD3^+^ T lymphocytes. E) Frequency of CD25^+^FoxP3^−^ cells within CD4^+^ T lymphocytes. F) Frequency of CD25^+^FoxP3^+^ cells within CD4^+^ T lymphocytes. G) Frequency of Ly6c^hi^ cells within CD11b^+^ monocytes. H) Frequency of CD68^+^CD11b^++^ cells within CD11b^+^ monocytes. * indicates p<0.05 and ** indicates p<0.01, Mann-Whitney test between Ncf1* and WT; differences between days 0 and 7 are indicated by a: p<0.05, aa: p<0.01, aaa: p<0.001; differences between days 7 and 14 are indicated by b: p<0.05, bb: p<0.01, bbb: p<0.001; differences between days 7 and 21 are indicated by c: p<0.05, cc: p<0.01, ccc: p<0.001; differences between days 14 and 21 are indicated by d: p<0.05, dd: p<0.01, ddd: p<0.001, all Mann-Whitney test. White bars Ncf1*, gray bars WT.

In order to establish whether there were systemic disease-related and Ncf1-mutation-related alterations in the frequency of different functional subsets of T cells (CD4^+^ and CD8^+^), NK cells, B cells and monocytes, these populations were characterized in the peripheral blood (Fig. 6).

At D0 the only significant differences between both groups observed in the blood were in CXCR4^+^ B cells and CD25^+^FoxP3^−^CD4^+^ effector T cells. At D7 Ncf1* and WT presented significant differences in the frequency of circulating activated CD11b^++^CD68^+^ monocytes, CXCR4^+^ and total CD19^+^ B cells. When comparing D7 to D0, there were significant differences in both groups for Ly6c^hi^ monocytes and total activated monocytes, and for IgM^−^IgD^−^CD19^+^ post-switch and CXCR4^+^ B cells. The WT blood at D7 had significantly more effector and total CD4^+^ T cells, CD25^++^FoxP3^+^CD4^+^ regulatory T cells (Treg), and total CD19^+^ B cells than at D0. At D14 the only significant differences between WT and Ncf1* mice were in the frequency of blood post-switch and CXCR4^+^ B cells. However, when comparing D14 to D7, the frequencies of activated monocytes, total B cells and effector CD4^+^ T cells in the blood of Ncf1* and WT were significantly different, whereas WT had significantly less Treg cells than at D7 and the Ncf1* had significantly less circulating total CD4^+^ T cells.

When comparing the leukocyte subsets between Ncf1* and WT mice at D21 there was a significant alteration in the frequency of activated monocytes. When comparing between D7 and D21 the Ncf1* mice presented significant changes in activated monocytes, CXCR4^+^ B cells and total B cells. The WT counterparts presented significant changes in activated monocytes, total B cells and Treg cells. Significant differences could also be seen between D14 and D21 in WT and Ncf1* Ly6c^hi^ monocytes and WT effector CD4^+^ T cells. Another important observation was that CD68^+^CD11b^+^ cells in Ncf1 mice did not increment after the second DSS treatment, as in WT mice.

No significant changes were observed for CD69^+^CD4^+^ and CD69^+^CD8^+^ T cells, CXCR5^+^CD4^+^ and CXCR5^+^CD8^+^ T cells, neither for the NK cell subsets.

### Quantification of cytokines and pLRRK2 production by colonic inflammatory infiltrates

**Figure 7:**
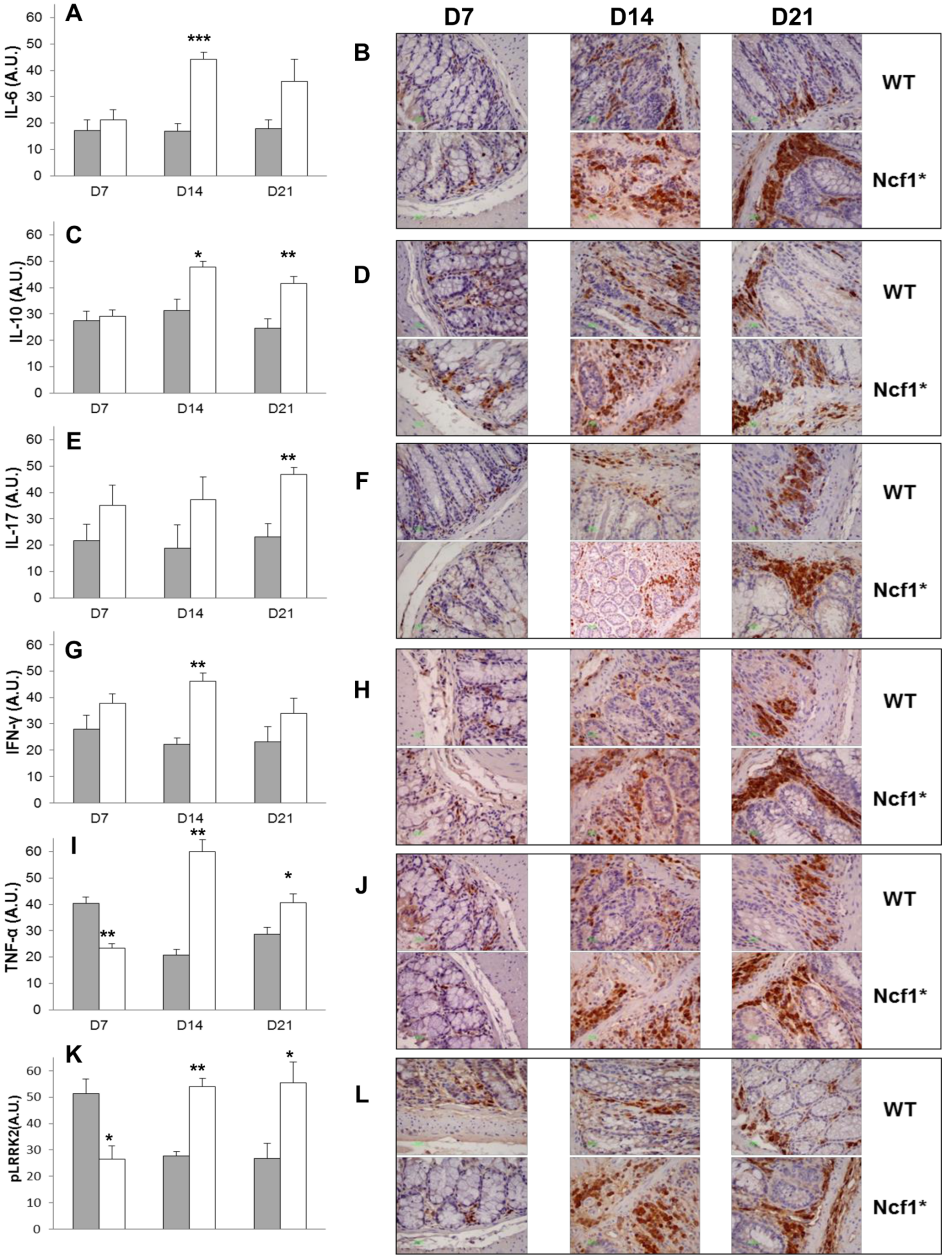
Colitis severity in Ncf1* mice results from increased local production of pro-inflammatory cytokines and LRRK2 phosphorilation. A) Relative expression of IL-6 in the inflammatory infiltrates of the colon mucosa at days 7, 14 and 21. B) Representative immunohistochemistry staining for IL-6 in the colon of WT and Ncf1* at days 7, 14 and 21. C) Relative expression of IL-10 in the inflammatory infiltrates of the colon mucosa at days 7, 14 and 21. D) Representative immunohistochemistry staining for IL-10 in the colon of WT and Ncf1* at days 7, 14 and 21. E) Relative expression of IL-17A in the inflammatory infiltrates of the colon mucosa at days 7, 14 and 21. F) Representative immunohistochemistry staining for IL-17A in the colon of WT and Ncf1* at days 7, 14 and 21. G) Relative expression of IFN-γ in the inflammatory infiltrates of the colon mucosa at days 7, 14 and 21. H) Representative immunohistochemistry staining for IFN-γ in the colon of WT and Ncf1* at days 7, 14 and 21. I) Relative expression of TNF-α in the inflammatory infiltrates of the colon mucosa at days 7, 14 and 21. J) Representative immunohistochemistry staining for TNF-α in the colon of WT and Ncf1* at days 7, 14 and 21. K) Relative expression of pLRRK2 in the inflammatory infiltrates of the colon mucosa at days 7, 14 and 21. L) Representative immunohistochemistry staining for pLRRK2 in the colon of WT and Ncf1* at days 7, 14 and 21. * indicates p<0.05, ** indicates p<0.01, *** indicates p<0.001 between Ncf1* and WT mice, Mann-Whitney test. White bars Ncf1*, gray bars WT. Magnification for all images 400×.

In order to evaluate whether local cytokine production could be involved in the higher disease severity in Ncf1* mice, we quantified the relative expression of IL-6, IL-10, IL-17A, IFN-γ and TNF-α in the inflammatory infiltrates of the colon after disease induction (Fig. 7). Since LRRK2 has been reported to be part of the susceptibility locus for CD [19], we equally quantified the relative expression of its phosphorylated form (pLRRK2) in the inflammatory infiltrates of the colonic mucosa on days 7, 14 and 21 in both groups (Fig. 7). No quantification was made for day 0 as there were no inflammatory infiltrates present.

At D7 TNF-α and pLRRK2 expression were significantly different between Ncf1* and WT mice (p = 0.002 and p = 0.016, respectively). Both groups differed significantly on D14 on the expression levels of IL-6 (p = 0.0005), IL-10 (p = 0.021), IFN-γ (p = 0.001), TNF-α (p = 0.002) and pLRRK2 (p = 0.008). Finally, at D21 expression levels between groups were significantly different for IL-10 (p = 0.011), IL-17A (p = 0.011), TNF-α (p = 0.036) and pLRRK2 (p = 0.030). To assess whether cytokine and pLRRK2 production in the colonic inflammatory infiltrates varied through the different induction and rest cycles within each group, their expression was compared between time-points. Thus, when comparing to D7, at D14 the Ncf1* colons expressed significantly more IL-6 (p = 0.004), IL-10 (p = 0.002) and TNF-α (p = 0.002). Comparing to D7, on D21 the colon infiltrates in Ncf1* mice expressed significantly more IL-10 (p = 0.013), TNF-α (p = 0.008) and pLRRK2 (p = 0.028); and comparing to D14 TNF-α (p = 0.014) and IFN-γ (p = 0.022) expression was significantly lower. On days D14 and D21, when comparing to D7, inflammatory infiltrates in WT colons expressed significantly less TNF-α (D14: p = 0.001; D21: p = 0.017) and pLRRK2 (D14: p = 0.02 and D21: p = 0.021).

### Serum cytokine quantification

**Figure 8:**
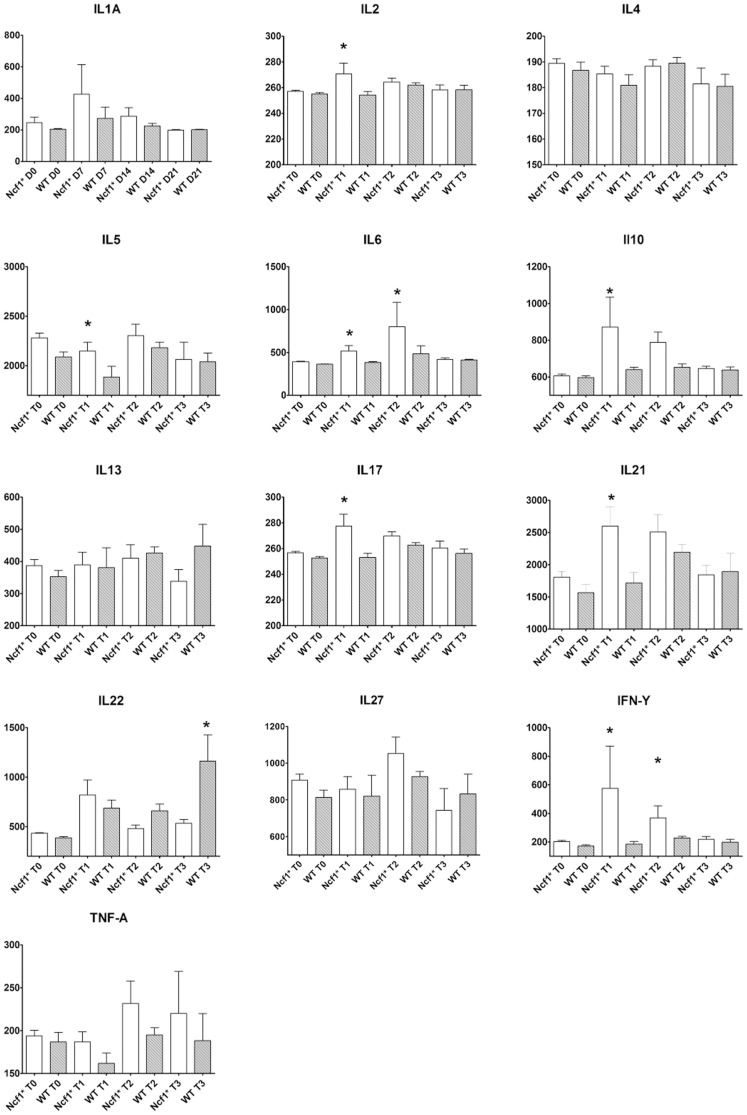
Variation of the serological cytokine titers in Ncf1* and WT mice during the different cycles. Data are presented as box-plots, where the boxes represent the 25th to 75th percentiles, the lines within the boxes represent the median, and the lines outside the boxes represent the 10th and 90th percentiles. For each group at D0 (n = 15), D7 (n = 15), D14 (n = 10) and D21 (n = 5); Asterisk indicates p<0.05, Mann-Whitney test between Ncf1* and WT. White bars Ncf1*, gray bars WT.

The serological levels of pro-inflammatory and anti-inflammatory cytokines were measured at different time-points, to assess whether the changes observed in the tissue had a systemic impact (Fig. 8).

At D0 cytokines titers were similar between the two groups, indicating that the *Ncf1*-mutation *per se* does not lead to aberrant cytokine production in naïve animals. For D7 the serum concentrations of IL-6, IL-17A, IL-22 and INF-γ significantly (p<0.05) increased in both groups when compared to D0 values, whereas Ncf1* mice also presented a significant (p<0.05) increase of IL-2 and IL-21. However, when comparing between both groups, Ncf1* mice had significantly higher titers of circulating IL-2, IL-6, IL-17A; IL-21 and INF-γ. At D14 the titers of IL-6, IL-17A, IL-21 and INF-γ in both groups still remained significantly (p<0.05) higher than the baseline values. Significant differences could be detected between both groups, namely Ncf1* mice had higher titers of IL-6, IL-10 and INF-γ than WT. At D21 the titers of IL-6 and IL-2 were still significantly (p<0.05) higher than baseline values in both Ncf1* and WT. IL-17A, IL-21 and INF-γ titers returned to baseline levels in both groups. WT mice had a significant increase in serum IL-22 when compared to Ncf1* mice, and IL-10 titers in the Ncf1* serum returned to baseline concentration.

## Discussion

Colitis is a major complication in CGD patients. However, existing experimental data linking defective ROS production and colitis development and severity are conflicting [16], [20], [21]. In particular, a suitable model to study how the deficient ROS-production by a defective NOX2 contributes to colitis severity is still missing. In the present study, we show, for the first time, that Ncf1* treated with repeated DSS-cycles intercalated by a resting period develop an earlier and severer colitis, contrasting to the milder colitis developed by oxidative-burst competent wild-type animals. This severe colitis in Ncf1* mice was mediated by increased local production of peroxynitrites, pro-inflammatory cytokines and phosphorylated LRRK2.

In wild-type mice from several strains, diarrhea and blood in stools are the consequence of crypt loss and mucosal erosions followed by inflammation in acute DSS-induced colitis, whereas repeated cycles of DSS administration intercalated by H_2_O result in chronic colitis, with areas of disease activity (inflammation) and inactivity, dysplasia, and epithelial hyperplasia [22], [23]. Thus, the clinical symptoms and histopathologic signs we observed in Ncf1* mice point towards a severe DSS-induced colitis. This Ncf1* colitis is characterized by the presence of inflammatory cells without epithelioid granulomata, and with dysplasia often with the criteria of severe dysplasia and intra-epithelial adenocarcinoma, in particular in the transversal region of the colon. This contrasted to the WT colitis which presented less severe morphological alterations considering both inflammation and epithelial dysplasia. Our data on Ncf1* mice are in accordance with what has been found in a systematic analysis of colon biopsies of CGD children –including p47^phox^-deficient- with chronic colitis: the inflammatory infiltrate present in the CGD colon and the extent of the disruption of the crypts, leading to functional changes, were less severe than in UC controls [24]. Moreover, since DSS is a chemically induced inflammation of the large bowel, which damages the mucosa and allows the fecal matter and therefore enteric bacteria across the mucosal barrier, it is possible that, similarly to CD and CGD, the Ncf1* mice have a failure in bacterial clearance leading to persistent bacterial/luminal contents in the bowel wall which may drive the chronic bowel inflammation. However, in CGD patients this incapacity to remove bacterial debris due to deficient phagocytosis leads to the formation of granulomata. In our study we did not find any granulomata in the colon mucosa, most probably because the colitis was not induced for long enough to form granulomata.

The time point at which T cells can be detected in the colon mucosa of DSS-induced colitis varies between studies and mouse strains [25]–[27]. The histological data we obtained at D7 suggest that the deficient ROS-production accelerates T cell infiltration of the colon mucosa in Ncf1* mice, which was accompanied by neutrophilic infiltration and a surge in local peroxynitrite production. Even though NO-production is one of the major pro-inflammatory effector mechanisms of myeloid cells, T cells –specially activated CD4^+^ T cells- are equally able of producing NO upon expression of inducible nitric oxide synthase (iNOS) [28]. Similarly to what has been reported for several experimental models of acute and chronic colitis, local NO-production drives an increased expression of TNF-α, IFN-γ and IL-6 [29]–[31]. Moreover, the inhibition of iNOS in *p47^phox−/−^* mice with DSS-induced colitis reduces disease severity by down-modulating the over-production of peroxynitrites [20]. In our study the exacerbated production of peroxynitrites in the colon mucosa of Ncf1* mice during the acute phase of disease might be responsible for the subsequent increase in local TNF-α, IFN-γ and IL-6 observed at D14 and continuing through D21. It has been previously shown that IL-10 production is needed to abrogate Th1 cells–mediated colitis serving as a mediator of regulatory T cell functions [32]. In our model we observed that the local and systemic levels of IL-10 were significantly higher in Ncf1* mice then in WT. However, this was not accompanied by an increased presence of Treg in the colon. Thus the observed surge in IL-10 might derive from other cells in an effort to down-modulate the markedly pro-inflammatory local and systemic cytokine profile, which is usually observed in the chronic phase of DSS-induced colitis [33]. Actually, we hypothesize that the higher B cell recruitment after the resting period relates to a peroxynitrite-induced IL-6 production by dentritic cells from the mucosa-associated lymphoid tissue, which triggers T cell-independent B cell migration [34].

The presence of significantly more CXCR4^+^ B cells in the peripheral blood of Ncf1* mice at baseline, D7 and D14 stresses that our animal model reproduces data obtained from IBD patients, in which circulating CXCR4^+^ B and plasma cells correlated with disease activity [35]. The absence of Mac-1^+^ macrophages at D7 in the colon of Ncf1* mice resembles what has been described for CGD patients, who present less CD68^+^ macrophages in their colon than CD patients and healthy individuals [36]. However, as they start infiltrating the colon mucosa after the acute phase –eventually facilitated by the vasodilatatory effect of NO-, these macrophages may become active players in the pro-inflammatory process by producing TNF-α and IFN-γ, which are hallmark cytokines of pro-inflammatory macrophages in colitis [25], [37]. Albeit no double immunohistological staining was done, we hypothesize that the exacerbated local TNF-α production in the Ncf1* colon mucosa results from the infiltrating macrophages.

The LRRK2 gene is located within the risk region associated with CD-susceptibility [19] and IFN-γ-induced LRRK2 expression in CD patients increases in the inflamed colonic mucosa. In our DSS-induced colitis model we observe that between D14 and D21 Ncf1* mice had a higher IFN-γ expression in the colon mucosa, which was paralleled by a higher expression of pLRRK2. Since LRRK2 induces NFAT expression [38], and exacerbated NFAT-expression associated with IL-17A production has been observed in human and mouse colitis [39], [40], we suggest that the surge of IL-17A in Ncf1* mice may be the result of a poor regulation of LRRK2-activity in the absence of ROS, thus stressing LRRK2-role in colitis susceptibility. Contrasting to previous reports [41], the increased local and systemic IL-17A-titers were not accompanied by higher systemic IL-1β-titers, but could be partially associated with the higher IL-21 serological concentration in Ncf1*, which stresses an abnormal T cell-activation.

Overall, our data present evidence that a normal production of ROS by a functional NOX2 is essential for the prevention of chronic inflammation leading to severe chronic colitis. The absence of ROS-production appears to promote peroxynitrites formation. The vasodilatatory properties of NO and its capacity to induce pro-inflammatory cytokines (and concomitant LRRK2 phosphorilation), seem to favor the infiltration and activation of leukocytes in the colon mucosa and feed a chronic inflammatory process. Since CGD is caused by point mutations in the NOX2 genes and the disease-associated colitis has a chronic profile, inducing two cycles of colitis with DSS intercalated by a resting period in mice with a point mutation in the *Ncf1* gene, the proposed colitis model presents an excellent model to study how the lack of oxidative burst can lead to the development of IBD with features reminiscent of CGD colitis.
